# A new species of *Leiochrides* Augener, 1914 (Annelida: Capitellidae) from the Beibu Gulf, South China Sea

**DOI:** 10.3897/BDJ.8.e59726

**Published:** 2020-11-19

**Authors:** Jun-Hui Lin, María E. García-Garza, He-Shan Lin, Jian-Jun Wang

**Affiliations:** 1 Third Institute of Oceanography, Ministry of Natural Resources, Xiamen, China Third Institute of Oceanography, Ministry of Natural Resources Xiamen China; 2 Universidad Autónoma de Nuevo León, Facultad de Ciencias Biológicas, Laboratorio de Biosistemática, San Nicolás de los Garza, Nuevo León, Mexico Universidad Autónoma de Nuevo León, Facultad de Ciencias Biológicas, Laboratorio de Biosistemática San Nicolás de los Garza, Nuevo León Mexico

**Keywords:** Polychaeta, Capitellidae, *
Leiochrides
*, South China Sea, taxonomy

## Abstract

**Background:**

Polychaetes of the family Capitellidae are poorly studied in Chinese waters. Amongst the known capitellid genera in China, *Leiochrides* Augener, 1914 is an unusual genus encountered in marine surveys.

**New information:**

In this study, a *Leiochrides* specimen was obtained during a survey conducted in the Beibu Gulf, northern South China Sea and described herein as a new species *Leiochrides guangxiensis* sp. nov. The new species differs from its congeners by having uniramous chaetiger 1, chaetigers 11–12 with notopodial capillaries and neuropodial hooks, abdominal hooks with seven teeth above the main fang in three rows, pygidium with four anal cirri, and branchial fascicles with up to 17 filaments. The taxonomic status of the monospecific genus *Pseudoleiocapitella* Harmelin, 1964 and *Leiochrides norvegicus* Fauchald, 1972 are discussed.

## Introduction

**Figure 1.**
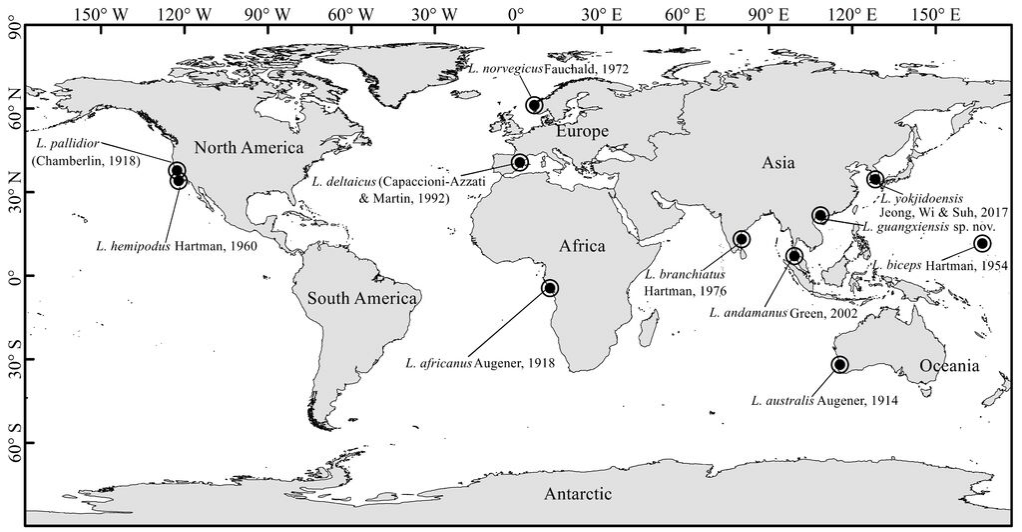
Type localities of all described *Leiochrides* species worldwide.

Polychaetes in the family Capitellidae resemble terrestrial earthworms (Rouse and Pleijel 2001). They are a group of organisms frequently encountered in marine surveys, especially in some highly-polluted habitats (Fauchald 1977). This family exhibits high generic diversity with 43 valid genera, including *Leiochrides* (García-Garza et al. 2019). *Leiochrides* was initially erected by Augener (1914) for the type species *L. australis* from West Australia, based on the following characters: thorax having 12 chaetigers with only capillaries; abdominal chaetigers with only hooks; transition between thorax and abdomen indistinct; and branchiae absent. The additional four described species agreed well with the original definition of *Leiochrides*, namely *L. africanus* from Congo (Augener 1918), *L. pallidior* from California (Chamberlin 1918), *L. biceps* from northern Marshall Islands (Hartman 1954), and *L. andamanus* from the Andaman Sea, Thailand (Green 2002). The definition of *Leiochrides* changed as new species were described. Hartman assigned two new species (namely *L. branchiatus* from the Indian Ocean and *Leiochrides* sp. from California) with one or two transitional chaetigers in posterior thorax to the genus (Hartman 1963, Hartman 1974), a unique character not found in previous reports. However, the new character was not accepted by Day (1967) and Fauchald (1977). Later, Blake (2000) and Green (2002) re-confirmed the presence of the transitional thoracic chaetigers in some *Leiochrides* species, and expanded the generic definition. The latest definition proposed by Magalhães and Blake (2017) followed Blake’s and Green’s, the genus being characterized as follows: chaetiger 1 uniramous or biramous; twelve thoracic chaetigers; chaetigers 1–10 with only capillaries; chaetigers 11–12 may have only capillaries or be transitional with notopodial capillaries and neuropodial hooks; branchiae present or absent. The emended definition of *Leiochrides* overlapped with that of *Pseudomastus* (Capaccioni-Azzati and Martín 1992), a monospecific genus from the Mediterranean Sea, as pointed out by Jeong et al. (2017). After morphological comparison between these two closely related genera, Jeong et al. (2017) considered *Pseudomastus* as a junior synonym of *Leiochrides*. To date, the genus *Leiochrides* contains 10 valid species worldwide, as shown in Fig. 1.

In China, capitellid polychaetes are frequently encountered in marine surveys. However, they are poorly understood, as most recorded species lack taxonomic descriptions and illustrations. Based on personal collections from southern China, more than 10 genera are recognized within this family, including *Barantolla*, *Capitella*, *Heteromastus*, *Leiocapitella*, *Leiochrides*, *Mediomastus*, *Notodasus*, *Notomastus*, *Parheteromastus*, and *Promastobranchus*. Of these, the genus *Leiochrides* is rarely collected, as well as *Leiocapitella* and *Parheteromastus*. *Leiochrides* specimens have been previously collected from Chinese waters, and they were usually identified as *Leiochrides australis* (Liu 2008). However, these species records need further confirmation, as *Leiochrides australis* was described from Australian waters. In this study, a complete *Leiochrides* specimen was collected from shallow soft sediment in the Beibu Gulf, northern South China Sea. It clearly differs from its congeners in a combination of morphological characters, and is described herein as a new species, *Leiochrides guangxiensis* sp. nov. Additionally, the taxonomic status of the monospecific genus *Pseudoleiocapitella* Harmelin, 1964 and *Leiochrides norvegicus* Fauchald, 1972 was found to be doubtful when reviewing the original descriptions and illustrations of capitellid species related to *Leiochrides*. The taxonomic relationship of the *Capitellethus*-*Leiochrides*-*Leiocapitella* complex needs a revision in the future.

## Materials and methods

The *Leiochrides* specimen was obtained from the Beibu Gulf, northern South China Sea in October, 2017. The sediment was characterized by high percentage of mud. Sediment samples were collected with a grab sampler (surface area 0.05 m^2^) and washed through a 0.5 mm sieve on board. All specimens retained by the sieve were fixed with 7% diluted formalin in seawater. In the lab, the *Leiochrides* specimen was transferred to 70% ethanol. The methyl green staining pattern (MGSP) was used to identify the distribution of glandular areas, following the protocol of Warren et al. (1994). The specimen was submerged in a solution of methyl green staining in 70% ethanol for 1 minute, then washed in clean 70% ethanol to eliminate excess stain. Light microscope images were obtained by means of a Leica M205A stereomicroscope equipped with a Leica DFC 550 digital camera. The structure of abdominal hooks was observed under a light stereomicroscope (Axio Imager Z2, ZEISS) using oil immersion. Scanning Electron Microscopy (SEM) observation was conducted on a middle fragment of the worm using a ZEISS SUPRA 55 SAPPHIRE at Xiamen University, following a process of dehydration, critical point drying, and gold coating.

The type material of the new species was deposited in the Third Institute of Oceanography, Ministry of Natural Resources, Xiamen, China.

## Taxon treatments

### Leiochrides guangxiensis

Lin, García-Garza, Lin, & Wang, 2020 sp. n.

76C6AAAD-B4C1-5EC6-990D-B68E727B1158

82795320-A115-442A-9DCF-FBDFD0D679DB

#### Materials

- **Type status:** Holotype. **Occurrence:** individualCount: 1; lifeStage: adult; **Taxon:** kingdom: Animalia; phylum: Annelida; class: Polychaeta; family: Capitellidae; genus: Leiochrides; specificEpithet: guangxiensis; **Location:** higherGeography: South China Sea; continent: Asia; waterBody: South China Sea; country: China; stateProvince: Guangxi Province; locality: the Beibu Gulf, off Guangxi Province; verbatimDepth: 12 m; verbatimLatitude: 21.626°N; verbatimLongitude: 108.6376°E; **Event:** samplingProtocol: Grab sampler; eventDate: 27-10-2017; habitat: muddy sediment; **Record Level:** institutionID: Third Institute of Oceanography, Ministry of Natural Resources; institutionCode: TIO-MNR; basisOfRecord: PreservedSpecimen

#### Description

**Figure 2.**
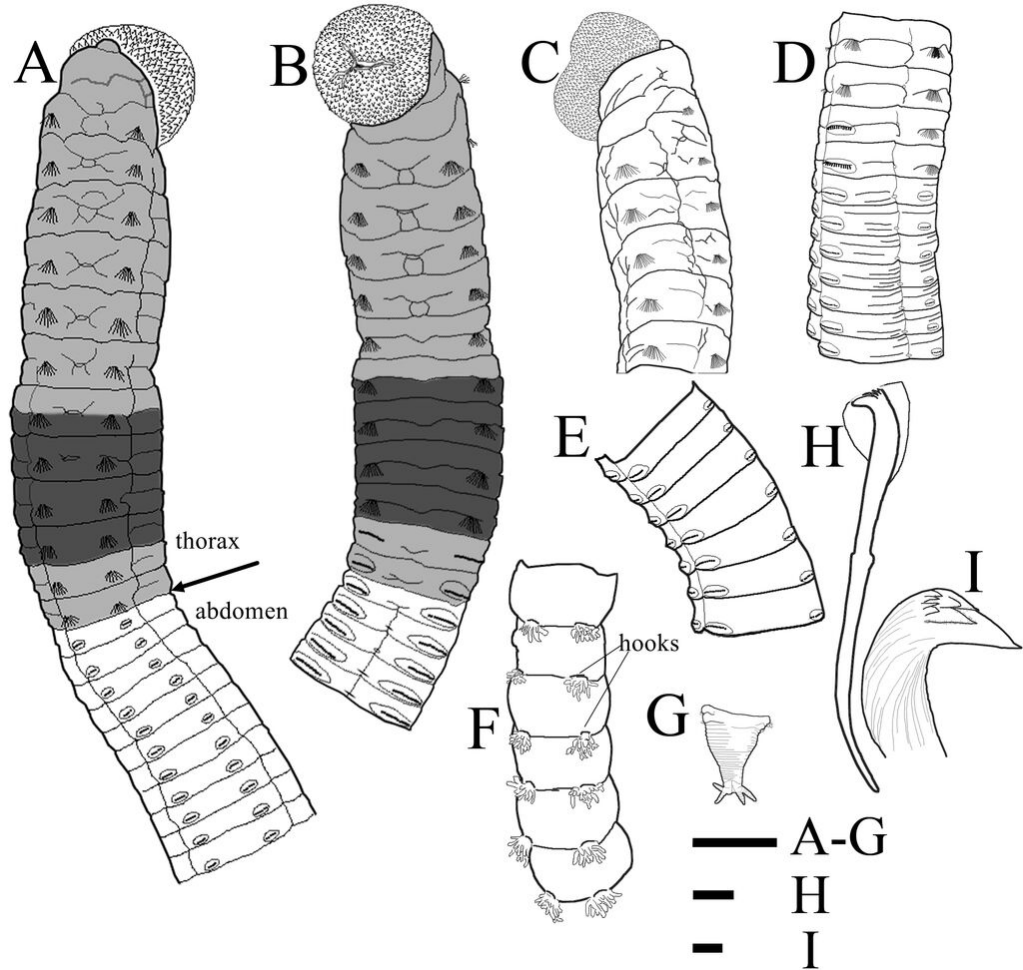
Holotype of *Leiochrides guangxiensis* sp. nov. **A.** thorax and anterior abdomen (23 chaetigers) in dorsal view, arrow indicates the separation of thorax and abdomen; **B.** thorax and anterior abdomen (17 chaetigers) in ventral view; **C.** anterior end in lateral view; **D.** chaetigers 9–19, showing transition between thorax and abdomen in lateral view; **E.** middle abdomen in lateral view; **F.** posterior abdomen in dorsal view; **G.** pygidium; **H.** hooded hook from middle abdomen; **I.** dentition of abdominal hooks. Shading on A–B indicates methyl green staining. Scale Bar: A–G = 1 mm; H = 10 μm; I = 2 μm

**Figure 3.**
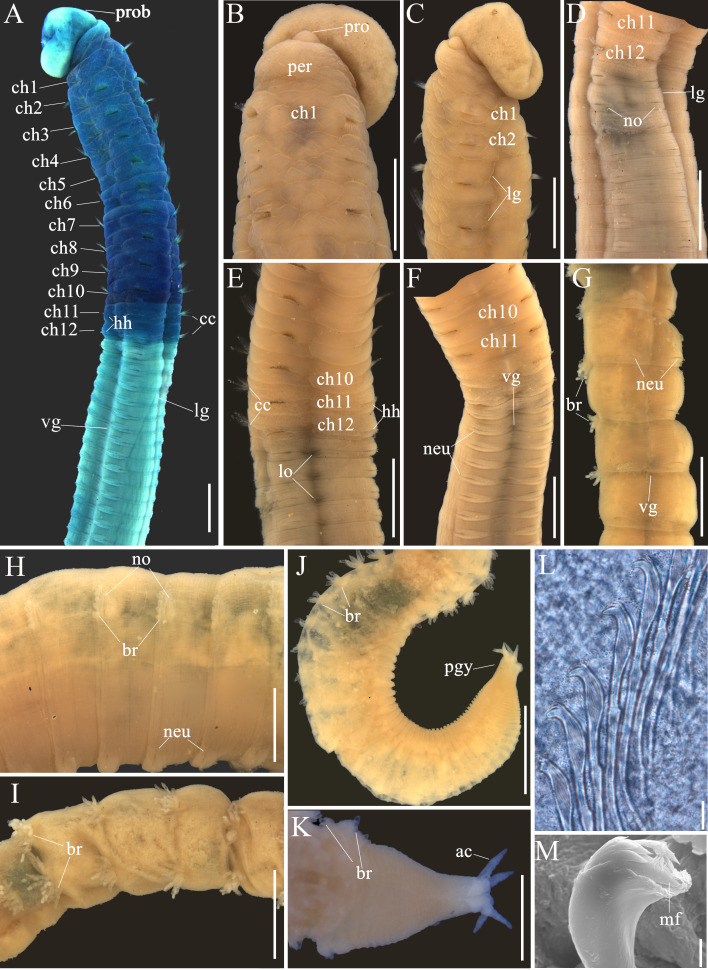
*Leiochrides guangxiensis* sp. nov. holotype. **A.** thorax and anterior abdomen in lateral view, showing methyl green staining; **B.** anterior end in dorsal view; **C.** anterior end in lateral view; **D.** chaetigers 11–20 in dorsal view; **E.** chaetigers 7–17 in lateral view; **F.** chaetigers 8–20 in ventral view; **G.** posterior abdomen; **H–J.** notopodial branchiae; **K.** posterior end showing branchiae and anal cirri; **L.** abdominal hooks; **M.** SEM photos of abdominal hooks. Abbreviations: ac, anal cirrus; br, branchiae; cc, capillary chaetae; ch, chaetiger; hh, hooded hook; lg, lateral groove; lo, lateral organ; neu, neuropodia; no, notopodia; per, peristomium; pro, prostomium; prob, proboscis; pyg, pygidium; vg, ventral groove. Scale Bar: A–J = 1 mm; K = 0.5 mm; L = 10 μm; I = 2 μm

Holotype (TIO-BTS-Poly-111) complete, but broken into four fragments. Anterior fragment (Fig. 2A) measuring 21.2 mm long by 1.7 mm wide for 40 chaetigers; two middle fragments with 25 chaetigers and 27 chaetigers, respectively; posterior fragment heavily coiled, measuring 63.1 mm long for more than 150 chaetigers. Color in alcohol tan on thorax (Fig. 3B) and whitish tan on abdomen. Prostomium short and conical, with blunt anterior end (Fig. 2A and 3B). Everted proboscis globular, with numerous minute papillae (Fig. 2B and 3B). Peristomium achaetous, wider than long, same length as chaetiger 1, but narrower (Fig. 2A and B). Eyespots not observed. Nuchal organs indistinct. Thorax areolated from peristomium to chaetiger 6 (Fig. 3B and C), weakly areolated on chaetigers 7–8, remaining segments smooth. Lateral groove distinct from chaetiger 2 to end of anterior fragment (Fig. 2A and 3A).

Thorax with an achaetous peristomium and 12 chaetigers (Fig. 2A, B and 3A). Chaetiger 1 uniramous, with only capillaries in notopodia (Fig. 2C). Chaetigers 2–10 with only capillaries in both rami (Fig. 3A). Chaetigers 11–12 transitional with notopodial capillaries and neuropodial hooks (Fig. 2D and 3E). Intersegmental grooves distinct in thorax, except between peristomium and chaetiger 1. Chaetigers 6–10 biannulate (more evident in lateral view), wider than long (Fig. 2A and B). Notopodia dorso-lateral in first chaetiger, gradually moving dorsally to end of thorax, and neuropodia ventro-lateral (Fig. 2A and B). Chaetal fascicles inserted just posterior to mid-line of thoracic segments (Fig. 2A and B). Notopodia of chaetigers 1–12 and neuropodia of chaetigers 2–10 each with 20–30 capillaries per fascicle; neuropodia of chaetigers 11–12 with approximately 50 hooks per fascicle. Thoracic hooks of similar shape to abdominal hooks, but shaft markedly longer. Lateral organs located between noto- and neuropodia, closer to notopodia in thorax and anterior abdomen, as small rounded pores (Fig. 3E); those in posterior abdomen indistinct. Genital pores not seen.

Transition between thorax and abdomen indistinct, marked by change in chaetal arrangement (Fig. 3A and E) and MGSP (Fig. 2A and B). Abdominal segments shorter than posterior thoracic chaetigers in anterior abdomen, tapering gradually to pygidium. Parapodial lobes slightly swollen in anterior abdomen (Fig. 3D and F), yet reduced in posterior abdomen (Fig. 3G and I). Notopodial hooks present from chaetiger 13 (first abdominal chaetiger). Notopodial lobes well separated throughout abdomen (Fig. 2A, B, E and F). From abdominal chaetiger 11, gap between notopodial lobes of same chaetiger becoming larger to end of anterior fragment (Fig. 2A). Chaetal fascicles positioned posterior to mid-segment in anterior abdomen (Fig. 3D and E), and near posterior edge of segment in posterior abdomen (Fig. 3G–I). Abdomen with hooks only, approximately 30 hooks per fascicle in notopodia and more than 100 hooks per fascicle in neuropodia.

Notopodial and neuropodial abdominal hooks similar along body, with long anterior shaft, developed shoulder, angled node, distinct constriction, and short hood (Fig. 2H); posterior shaft longer than anterior one, attenuated to terminal end (Fig. 3L). Hooks (Fig. 3M) with three rows of teeth above main fang: two large teeth in basal row, a larger median tooth, and four smaller teeth above basal teeth. Main fang subtriangular, longer than wide (Fig. 2I).

Notopodial branchiae present in posterior abdomen, located on posterior edge of segment, may be retractile (Fig. 3H–K). At first, notopodia with 3–6 papillary branchiae (Fig. 3H); towards the pygidium, the branchial lobes gradually increasing in number and length, and the largest branchial fascicle with up to 17 finger-shaped filaments (Fig. 2F); then decreasing in number to end of the body (Fig. 3J and K). More than 10 achaetous segments without branchiae before pygidium (Fig. 2G). Pygidium with four digitate anal cirri (Fig. 3J and K)

*Methyl green staining*: Dark blue stain from post-chaetal part of chaetiger 7 to chaetiger 10 (Fig. 2A); medium blue stain from peristomium to pre-chaetal part of chaetiger 7, as well as on chaetigers 11–12. Abdominal region without any distinct staining pattern (Fig. 3A).

#### Etymology

The specific name is derived from the type locality, Guangxi Province.

#### Distribution

Currently known from the Beibu Gulf, northern South China Sea.

#### Ecology

The new species inhabits shallow subtidal waters where sediment is characterized by high percentage of mud.

#### Taxon discussion

The taxonomic and ecological knowledge of this genus is very rare in China, although *Leiochrides* species have been previously recorded (Liu 2008). This genus is characterized by having 12 thoracic chaetigers, of which the last one or two thoracic chaetigers may only have capillaries or be transitional with notopodial capillaries and neuropodial hooks (Magalhães and Blake 2017). Our specimen in the present study agrees well with the generic diagnosis. Morphological characters used for the separation of species within this genus include the following: the number of transitional thoracic chaetigers, the shape of first chaetiger, the dentition of abdominal hooks, the number of anal cirri, and the presence/absence of branchiae on posterior abdomen. Amongst all described *Leiochrides* species worldwide, *L. guangxiensis* sp. nov. is closely similar to *L. branchiatus* Hartman, 1976 from the Bay of Bengal and *L. deltaicus* (Capaccinoi-Azzati & Martin, 1992) from north-western Mediterranean Sea in having the last two thoracic chaetigers transitional with notopodial capillaries and neuropodial hooks, uniramous chaetiger 1, and the presence of notopodial branchiae on posterior abdomen. However, *L. guangxiensis* sp. nov. differs from *L. branchiatus* in that abdominal hooks of the new species have seven teeth in three rows above main fang instead of three teeth in triangular arrangement as in *L. branchiatus*. *L. guangxiensis* sp. nov. can also be distinguished from *L. deltaicus* in the maximum number of branchial filaments per fascicle and the number of anal cirri. *L. guangxiensis* sp. nov. bears branchiae with up to 17 branchial filaments per fascicle and pygidum with four anal cirri, whereas in *L. deltaicus*, posterior abdomen has 2–4 branchial lobes per fascicle and pygidium with three anal cirri. As for MGSP, the new species stains dark blue from the postchaetal area of chaetiger 7 to chaetiger 10, medium blue on the remaining thoracic chaetigers, and light green staining on the abdomen, these characters being distinct from those of other *Leiochrides* species.

The dentition of abdominal hooks is widely used in capitellid taxonomy, as their ultrastructure is highly specific (Hartman 1947). The characteristic feature of the genus *Leiochrides* is that abdominal hooks bear two larger teeth in the basal row above the main fang, although the number of small teeth may vary amongst *Leiochrides* species. Green (2002) pointed out that this mentioned character is conservative in *Leiochrides* species, as also present in species of *Capitellethus* and *Leiocapitella*. These three genera show similarities in general appearance and the difference amongst these genera is in relation to the number of thoracic chaetigers (Magalhães and Blake 2017). In addition to one or two transitional chaetigers, the structural similarity of basal teeth in abdominal hooks amongst these three genera (*Capitellethus*-*Leiochrides*-*Leiocapitella* complex) indicates that they might have a close taxonomic relationship, which should be reviewed as suggested by Green (2002) and Magalhães and Blake (2017). Molecular data should be incorporated to help verify their phylogenetic relationship in the future.

In this study, the taxonomic status of the genus *Pseudoleiocapitella* Harmelin, 1964 and *Leiochrides norvegicus* Fauchald, 1972 was found to be doubtful when reviewing the literature on capitellid species related to *Leiochrides*. The monospecific genus *Pseudoleiocapitella* was established by Harmelin (1964) for *P. fauveli* (see pp 90–92, Pl.Ⅺ, figs. 1–7 in Harmelin 1964), characterized by the uniramous chaetiger 1, chaetigers 1–10 with only capillaries, chaetigers 11–12 with notopodial capillaries and neuropodial hooks, and the absence of branchiae. The chaetal arrangement of *Pseudoleiocapitella fauveli* matches the expanded generic definition of *Leiochrides* proposed by Magalhães and Blake (2017), indicating it might be a *Leiochrides* species. *Leiochrides norvegicus* was described by Fauchald (1972), based on specimens from the deep Sognefjorden off western Norway. Fauchald (1972) assigned this species to the genus *Leiochrides*, due to the thorax with 12 chaetigers. However, *L. norvegicus* has the first two abdominal chaetigers with notopodial capillaries and neuropodial hooks, which did not match the generic diagnosis of *Leiochrides*. Observed from its chaetal arrangement in the anterior part, *L. norvegicus* actually bears 14 instead of 12 thoracic chaetigers, of which the last two thoracic chaetigers are transitional. If so, *L. norvegicus* should be a *Leiocapitella* species rather than a *Leiochrides* species. In addition to the number and location of transitional chaetigers, other morphological characters of *L. norvegicus* have also been found in the members of *Leiocapitella*, such as uniramous chaetiger 1 and the presence of branchiae on posterior abdomen (Green 2002, Magalhães and Blake 2017). According to the above analysis, the genus *Pseudoleiocapitella* Harmelin, 1964 and *Leiochrides norvegicus* Fauchald, 1972 need a revision, and it would be necessary to examine the type materials of *Pseudoleiocapitella fauveli* and *Leiochrides norvegicus*.

## Supplementary Material

XML Treatment for Leiochrides guangxiensis
